# A Study of Marital Satisfaction Among Non-Depressed and Depressed Mothers After Childbirth in Jahrom, Iran, 2014

**DOI:** 10.5539/gjhs.v7n3p140

**Published:** 2014-11-25

**Authors:** Marzieh Kargar Jahromi, Azam Zare, Mahboobeh Taghizadeganzadeh, Afifeh Rahmanian Koshkaki

**Affiliations:** 1 Community Health Nursing, Faculty Member, Jahrom University of Medical Science, Jahrom, Iran; 2 Medical-Surgical Nursing, Shiraz University of Medical Science, Shiraz, Iran; 3 Medical-Surgical Nursing, Jahrom University of Medical Science, Jahrom, Iran

**Keywords:** marital satisfaction, depression, childbirth, Iran

## Abstract

**Introduction:**

Birth is one of the most wonderful events in nature and pregnancy and delivery are major developments for most married women. Similar to the pregnancy period, the period of time following delivery is accompanied by certain mental and physical changes in women. During this time, mothers experience a full range of mental disorders, varying from minor to psychotic. The objective of this study was to examine marital satisfaction among non-depressed and depressed mothers who visited primary health centers in Jahrom after childbirth in 2014.

**Method and Material:**

This is a descriptive cross-sectional study. The study population consisted of 80 mothers, who were in the 6 to 12 weeks of delivery and had visited primary health centers in Jahrom from April to July, 2014.To select the participants, the researcher looked thorough the files at each center and chose the mothers who were qualified for the study based on convenience sampling. The criteria for participation were: being aged from 20 to 40; being in the 6-12 weeks since delivery; having a healthy newborn; willingness to participate in the study. The participants were divided into the two groups of mothers suffering from postpartum depression (40 women) and mothers not affected by postpartum depression (40 women) on basis of questionnaire. The study follows the ethics in a scientific study. The researcher personally visited the primary health centers and explained the objectives of the study to the participants. Subsequently, the participants were asked to complete a demographic questionnaire, Enrich Marital Satisfaction Scale, and Edinburgh Postpartum Depression Scale. The participants were allowed one hour to complete the questionnaires.

**Result:**

The results showed that the average age of depressed and non-depressed women was respectively 28.1±5 and 29.4±5.5. Regarding the sex of the newborns, 53% of the depressed women had a son and 46.7% had a daughter. In the non-depressed group, 43.3% of the mothers had a son and 56.7% had a daughter. 56.7% of the depressed mothers were first-time mothers; however, 43.3% of the non-depressed mothers had experienced childbirth for the first time.

Most of the women in both groups had a high-school diploma—53% of the depressed mothers and 51% of the non-depressed. 66.7% of the depressed mothers had had natural childbirths; 60% of the non-depressed mothers had had Cesareans. There was not a statistically meaningful difference between the two groups in terms of the demographic variables. The average depression score of the depressed group was 13.7 with a standard deviation of 3.2; the average depression score of the non-depressed group was 5.8 with a standard deviation of 2. There was a statistically significant difference between the two groups in terms of marital satisfaction.

**Conclusion:**

Postpartum depression is a major and common health problem, affects many women after childbirth and inflicts not only direct costs on the health care system, but causes extensive indirect losses due to mothers’ inability to function.

Though this condition is prevalent among new mothers, not many researchers have addressed it in small towns and investigated its relationship with marital satisfaction. In addition, most women suffering from postpartum depression know very little about the disorder. Accordingly, it is vital to educate women and conduct more studies on the issue.

## 1. Introduction

Birth is one of the most wonderful events in nature (Ivanbagha & Malakuti, 2005), and pregnancy and delivery are major developments for most married women. Word Health Organization (WHO) recognizes women’s hygienic needs as one of its top priorities. Women, in addition to comprising half of the population of the world, have an important influence on the health of the next generations (Nick-Bakht & Davazdahemami, 2003). Similar to the pregnancy period, the period of time following delivery is accompanied by certain mental and physical changes in women. During this time, mothers experience a full range of mental disorders, varying from minor to psychotic (Brockington, 2004). Such disorders may be due to the return of an old mental illness or a whole new disorder (Simbar, Azgoli, Pazndeh, Jansary, & Khodakarami, 2005). Postpartum mental disorders fall into three groups: postpartum sadness, postpartum depression, and postpartum psychosis (Kaplan & Sodok, 2005).

Sadness, a self-restrictive and temporary condition, is a very common behavioral disorder in the postpartum period and affects 50% to 80% of women (Khodadadi et al., 2009; Kheirabadi et al., 2010). However, in some cases, women suffer from postpartum depression, which is a serious and enduring form of depression and often appears in the fourth week after childbirth (Khodadadi, Mahmoudi, & Mirhaghjou, 2009).

Postpartum depression is characterized by melancholy, dissatisfaction with life, irritability, insomnia, recurrent dizziness, mental bipolarity, antagonism toward the baby, failure to protect the baby, and suicidal thoughts (Kaplan & Sodok, 2005). A major and common health problem, postpartum depression affects many women after childbirth and inflicts not only direct costs on the health care system, but causes extensive indirect losses due to mothers’ inability to function (Ivanbagha, & Mohamd-Alizadeh, 2004; Gelder, Mayou, & Geddes, 2005). The frequency of this condition varies, which is due to the role of environmental factors (Tannous, Gigante, Fuchs, & Busnello, 2008). The frequency rate of postpartum depression in Iran is similar to other developing countries, which is three times greater than in developed countries (Janani, Saki, & Chngavy, 2005).

The pathology of postpartum depression is highly controversial. In order to successfully adapt to pregnancy and childbirth, a woman needs to have physical, intrapersonal, family and communicative compatibilities (Aghapour & Mohammadi, 2011). Marital satisfaction is a mental perception and has a major impact on a woman’s adjustment to what happens during and after pregnancy. In this study, it is assumed that women who are affected by postpartum depression have not had marital satisfaction, and are, consequently, more vulnerable to the occurrences that can result in mental and emotional complications.

Mothers suffering from depression have difficulty interacting with their newborns (Posmontier, 2008). Such poor interactions can damage newborns’ cognitive developments (Cornish, Mihin, & Barnett, 2005; Diego, & Hernandez, 2005; in award, 2005). Furthermore, postpartum depression can adversely affect the relationship between a woman and her husband and depress the husband; if left untreated, postpartum depression may result in separation or divorce (Kaplan & Sodok, 2005).

A fulfilling marital relationship is essential to a family’s healthy performance and affects effective parenting both directly and indirectly. It also improves interaction among children and between children and their parents and enhances competence and adaptability in children (Khoda-Rahimi, 2003).

It should be noted that failure to diagnose depression often occurs at primary health centers, the most popular places to receive health care (Cunningham et al., 2005). Postpartum depression, which harms both families and societies, is a multifactorial disorder; however, through proper education, we can identify the factors affecting postpartum depression at clinical centers and take steps to prevent, cure, and eventually reduce the occurrence of such depression and the harms resulting from it. Accordingly, a better-informed staff can provide better care—after all; these people are at the frontline of providing care to mothers. The objective of this study was to examine marital satisfaction among non-depressed and depressed mothers who visited primary health centers in Jahrom after childbirth in 2014.

## 2. Materials and Method

### 2.1 Setting

This is a descriptive cross-sectional study. The study population consisted of mothers, who were in the 6 to 12 weeks of their delivery and had visited primary health centers in Jahrom from April to July, 2014.

### 2.2 Data Collection

1) Edinburgh Postpartum Depression Scale (EPDS)

The 10-item questionnaire was designed by Cox in 1987 to determine the extent of postpartum depression (Cox, Holden, & Sagovsky, 1987). There are four possible answers for each of the ten questions which are assigned a score from 0 to 3. The division score in this study was 12, meaning the women whose scores were 12 or above were considered as depressed and those with scores below 12 were considered as normal. Iranian version of the questionnaire was acceptable, valid and reliable instrument for measuring postpartum depression (Mazhari & Nakhaee, 2007).

2) Enrich Marital Satisfaction Scale

The questionnaire was developed by David H. Olson, David J. Fournier, and Joanne M. Druckman. In this study, the 115-item questionnaire was employed. The questionnaire is used to identify potential conflicts, strengths and growth areas in a marriage. It is also used to identify couples who are in need of consultation and strengthening their relationship. Enrich is a reliable tool widely used to evaluate marital satisfaction. It consists of 14 sub-scales. The first scale includes 5 questions, and the rest include 10. The sub-scales in the questionnaire are: ideal alteration (1-5); marital satisfaction (6-15); personality compatibility (16-25); relationship (26-35); conflict resolution (36-45); financial management (46-55); free time activities (56-65); sexual relationship (66-75); children and parenting (76-85); family and friends (86-95); equality (96-105); religious tendencies (106-115).

The 115-item questionnaire used in Iran, each question was assigned 5 choices. The choices were: extremely; very; somewhat; a little; very little. Except for questions 96 to 105 whose possible answers were “always,” “often,” “I’m not sure,” “rarely”, and “never,” the other questions were scored from 0 to 4. Accordingly, the highest possible score on this questionnaire was 460. Higher scores indicated greater marital satisfaction.

### 2.3 Intervention

To select the sample, 8 primary health centers were chosen on a simple random sampling. The study follows the ethics in a scientific study. To select the participants, the researcher looked thorough the files at each center and chose the mothers who were qualified for the study based on convenience sampling. The researcher personally visited the primary health centers and explained the objectives of the study to the participants. The criteria for participation were: being aged from 20 to 40; being in the 6-12 weeks since delivery; having a healthy newborn; willingness to participate in the study. The participants were divided into the two groups of mothers suffering from postpartum depression (40 women) and mothers not affected by postpartum depression (40 women) on basis of questionnaire. In total, there were 80 participants in the study. Subsequently, the participants were asked to complete a demographic questionnaire, Enrich Marital Satisfaction Scale, and Edinburgh Postpartum Depression Scale. The participants were allowed one hour to complete the questionnaires.

### 2.4 Data Analysis

To analyze the results of the study, the data collected in the questionnaires was saved on a computer and the statistical software SPSS version 16 was used. To analyze the findings, the researcher used descriptive tests—frequency and frequency percentage, mean, and standard deviation—the analytical test of T-test.

## 3. Results

The results showed that the average age of depressed and non-depressed women was respectively 28.1±5 and 29.4±5.5. Regarding the sex of the newborns, 53% of the depressed women had a son and 46.7% had a daughter. In the non-depressed group, 43.3% of the mothers had a son and 56.7% had a daughter. 56.7% of the depressed mothers were first-time mothers; however, 43.3% of the non-depressed mothers had experienced childbirth for the first time.

Most of the women in both groups had a high-school diploma—53% of the depressed mothers and 51% of the non-depressed. 66.7% of the depressed mothers had had natural childbirths; 60% of the non-depressed mothers had had Cesareans. There was not a statistically meaningful difference between the two groups in terms of the demographic variables. The average depression score of the depressed group was 13.7 with a standard deviation of 3.2; the average depression score of the non-depressed group was 5.8 with a standard deviation of 2. The average marital satisfaction scores and their sub-scales for the both groups are included in Table 2. The differences between the average marital satisfaction scores of the two groups are presented in Table 3.

**Table 1:** Frequency distribution and descriptive statistics of the participants based on the variable of marital satisfaction (the sum of the sub-scales)

Group	Level of satisfaction	Frequency	Percentage
**Depressed**	Low	24	60
	Moderate	14	35
	High	2	5
	Total	40	100
**Non-depressed**	Low	16	40
	Moderate	10	25
	High	14	35
	Total	40	100

**Table 2:** Mean of marital satisfaction and the related sub-scales in the two groups

Marital satisfaction sub-scales	Group	Mean	Standard Deviation
**Ideal alteration**	Depressed	9.7	3.1
	Non-depressed	13.7	2.8
	**Total**	11.5	3.6
**Marital satisfaction**	Depressed	18.9	6
	Non-depressed	25.6	3.8
	**Total**	22	6
**Relationship**	Depressed	24.8	6.8
	Non-depressed	20.2	6.6
	**Total**	22.6	7
**Free time activities**	Depressed	20.2	7.2
	Non-depressed	28.8	5.8
	**Total**	24.2	7.8
**Personality compatibility**	Depressed	21.2	8.6
	Non-depressed	28.2	5.9
	**Total**	24.4	8.2
**Conflict resolution**	Depressed	19	4.6
	Non-depressed	24.3	6.8
	**Total**	21.5	6.3
**Financial management**	Depressed	28.7	6
	Non-depressed	29.9	3.3
	**Total**	29.3	4.9
**Sexual relationship**	Depressed	26.4	5.9
	Non-depressed	29.4	6
	**Total**	27.8	6
**Parenting**	Depressed	18.5	4.7
	Non-depressed	26	11.8
	**Total**	22	9.4
**Family and friends**	Depressed	18.6	5.8
	Non-depressed	12.3	4.7
	**Total**	15.7	6.1
**Equality**	Depressed	24.6	5.1
	Non-depressed	19.8	6.3
	**Total**	22.4	6.1
**Religious tendencies**	Depressed	21	4.7
	Non-depressed	19.9	5
	**Total**	20.5	4.8
**Marital satisfaction (Total)**	Depressed	251	33.3
	Non-depressed	278.7	26.7
	**Total**	263.9	33

**Table 3:** The mean difference of marital satisfaction scores in the two groups

Group	Marital Satisfaction	Mean	Standard Deviation	t	Degrees of freedom	P-Value
**Depressed**	Low satisfaction	16.2	4	2.7	14	0.016
	High satisfaction	11.8	1			
**Non-depressed**	Low satisfaction	7	1.4	0.936	12	0.368
	High satisfaction	5.5	2.1			

## 4. Discussion

Based on the data collected by Enrich Marital Satisfaction Scale, 60% of the women in the depressed group had low marital satisfaction, and 35% had moderate satisfaction. In comparison, 40% of the non-depressed women had low marital satisfaction and 35% had high marital satisfaction. There was a statistically significant difference between the two groups in terms of marital satisfaction.

These findings consistent with the results of the study of Aghapour and Mohammadi (2011) where postpartum depression was compared in working women and stay-at-home housewives and the influences of social support and marital compatibility were investigated. These findings also consistent with the study of Hadizadeh, Bahri and Tavakolizadeh (2004), a comparison of the occurrence of postpartum depression in first-time mothers who had had natural childbirths and those who had had emergency cesareans, the results of which showed that there was a relationship between the participants’ postpartum depression scores and their marital satisfaction.

Each issue can become contentious conflict in couple’s relationships. Depressed mothers have described postpartum depression as go to the gates of Hell and worst nightmares. Postpartum depression creates many problems in family relationships, mental health and its relationship with the wife’s family. Depressed women tend to exaggerate their problems and to reduce their relevance to family members more than ever. But they need more support (Aghapour & Mohammadi, 2011).

In other studies, Shabani et al. (2008) and Jafarpoor (2007) examined the relationship between the frequency of postpartum depression and stressful events in life in 975 pregnant women who visited the primary health centers in Kermanshah, Iran. They concluded that from among the stressful events in question, frequent quarrels with the husband (P<0.001), trouble with the husband’s family (P=0.001), the beginning or end of an academic period (P=0.015), divorce (P=0.026), and changes in bedtime (0.049) had the closest relationship with postpartum depression; the researchers also discovered a meaningful relationship between the frequency and severity of stressful events and postpartum depression (P<0.001).

In a study entitled “The relationship between postpartum depression and certain psychological-social factors in women in Kermansha, Iran”, Mousavi et al. (2008) studied 204 women and concluded that the husband’s behavior, a close relationship with the family and not having a history of depression can reduce the likelihood of postpartum depression.

The results of the present study are in agreement with the study of Kiani, Khadivzadeh, Sar-Golzaei, and Behnam, (2010) which investigated the relationship between marital satisfaction during pregnancy and postpartum depression. Similarly, the results agree with the findings of the study of Khodadadi, Mahmoudi, and Mirhaghjou, (2009) on the relationship between postpartum depression and mothers’ psychological-social situations. In their study of the influence of demographical characteristics on postpartum depression, Salehi, Tavafiyan, and Salehi, (2010) concluded that there was a relationship between marital satisfaction and postpartum depression. Thus, it is arguable that women affected by postpartum depression have less marital satisfaction compared to non-depressed women, and by enhancing marital satisfaction, the likelihood of postpartum depression will decrease significantly.

All researchers agree that a conscious effort to acquire the necessary skills to live life, especially during pregnancy could prevent potentially damage such as postpartum depression.

## 5. Conclusion

A review of the studies conducted in the area of postpartum depression—especially in Iran—shows that, though this condition is prevalent among new mothers, not many researchers have addressed it in small towns and investigated its relationship with marital satisfaction. In addition, most women suffering from postpartum depression know very little about the disorder. Accordingly, it is vital to educate women and conduct more studies on the issue.

It can be concluded that marital satisfaction affects the likelihood of women’s suffering from postpartum depression: women who have more marital satisfaction are less likely to be affected by postpartum depression. Limitations of the study as fallowing:

1) Since the participants had to be 4-6 weeks postpartum, qualified participants were hard to find.
2) This study was limited to new mothers in Jahrom, Iran, and the findings of the study should be extended with care.
3) This study was limited to women living in the town; in view of the differences in their cultures and environments, women living in rural areas may experience different levels of postpartum depression and marital satisfaction.
